# Institution for Feeble-Minded

**Published:** 1875-06

**Authors:** 


[From the Jacksonville Daily Journal, April 10th.]

### THE FEEBLE-MINDED.

This very worthy institution has been deservedly popular with the Twenty-Ninth General Assembly. Its economical administration has been so marked that the appropriations asked for ordinary expenses, by its Board of Trustees, were recommended in full by both the Governor and State Board of Public Charities, and was not reduced by any committee of the Legislature ; and the bill passed the house on Wednesday, by 120 ayes and no nays.

This was the only appropriation bill which passed the House this session with no dissenting voices.

The bill appropriating $175,000 for a new building for this Institution, also $10,000 for land, has also passed both Houses, and been approved by the Governor. On its final passage in the House, it received 90 ayes, several of its friends being absent. It is to be hoped that a building may speedily be erected, in order that the unsafe tinder-boxes now occupied may be vacated, and the pupils sheltered in safe and convenient buildings at an early day.

### ELOQUENT SPEECH.

During the discussion on the bill to appropriate money to erect buildings for the Feeble-Minded, the Hon. E. Callahan, of Crawford county, made the following eloquent speech :

Mr. Speaker : The gentleman from Whiteside has spoken strongly against the passage of this bill, and made his appeal to this reform legislature to rally at this point to stop the flow of money which we have been voting out of the State Treasury. Why this rally in favor of economy should be made when the feeble-minded children of the State, without reason enough to guide and care for themselves, are pressing their plea for charity, I cannot tell. I am prepared to respond to any proper appeal for economy in the expenditure of public money. I am as careful as any member on this floor about voting for appropriations of a questionable character. I would throw around the treasury all proper safeguards. I believe in taking care of money. But, sir, in civilized countries there are higher duties and more sacred obligations than hoarding or saving money. One of these duties is to care for the unfortunate who, through physical or mental disability, are unable to care for themselves. History tells us that in the distant past there were nations who relieved themselves from the support of these unfortunates by destroying their lives. Sometimes they were directly and openly killed; sometimes left in exposed positions to perish by the vicissitudes of the weather, by hunger and thirst, or to be destroyed by wild beasts. Civilized nations reject the theory of these barbarians—deny the right to destroy life, and accept the duty of care and support. It seems to me that gentlemen who vote against this bill are proposing to go back to the barbarian theory, and leave these poor children, in whose darkened minds the brightest flashes of reason are but as the twilight, unguided, unprotected, uncared for, to perish after suffering all the refinements of cruelty which are born of neglect. I was but recently a visitor at this institution. I never saw anything that so strongly moved my pity. I was astonished at its success in the education of these poor children, in whose minds reason is but a glimmering spark, and not a guiding star. Dr. Wilbur and his assistants have succeeded beyond expectation in their efforts to strengthen and direct their minds. They may never become valuable members of society, but if they only learn to care for themselves, the benefit repays the outlay. I will not attempt argument—though arguments are numerous. Every tongue and voice of humanity pleads for them, and I accept the plea and shall vote for the appropriation. I will say further that I fully concur with the gentleman from Bureau (Mr. Herron) that the building in which these children are kept is a disgrace to the State. It is an old wooden building, as combustible as a tinder-box. If t should take fire, the escape of the inmates would be next to impossible. They should not be kept in this peril of life a single hour longer than is absolutely necessary to provide other and safer quarters for them. The State cannot longer neglect to make this provision without subjecting its good name to reproach. Let us make this application and found this public charity, and practice our economic policy in measures where the unfortunate and the helpless are not so deeply concerned.

---

### A DESERVED COMPLIMENT.

(From the "Spectator.")

"The admirable work of Dr. J. Adams Allen, called 'Medical Examinations for Life Insurance,' and published by the Spectator Company, has now been five years before the insurance public in America; and the rapid sale of many successive editions has shown that its merits are highly appreciated here. But as it is especially adapted to American methods of doing business, and to the common American forms of application for a life policy, it was not designed for a foreign market, and probably its author never anticipated for it a circulation in Europe.

"But a book now comes to us from Berlin, entitled 'Der Gesellschaftsarzt; Handbuch fur Lebensversicherer; Herausgeber, Dr. A. F. Elsner,' which is highly recommended as the best manual for medical examinations accessible to German physicians. On a careful examination of this work, we find thirty-seven pages occupied with a very good glossary of medical terms; eighteen more devoted to an index; and the remainder filled with an almost literal translation of Dr. Allen's work, embodying the whole of it, except that the appendix is omitted and the preface condensed.

"Dr. Elsner is the editor-in-chief of the Berlin 'Versicherungs-Zeitung,' and is a gentleman of such high repute and responsibility in his own country, that his name is a guaranty of excellence. Had he merely published Dr. Allen's work with his approval, the compliment would have been a very high one. But he has taken the entire work, and 'made it his own' by displacing Dr. Allen's name from the title page and setting his own name there; surely, the most emphatic act of approval and eulogy that ever an author conferred on another. Dr. Elsner's willingness to vouch for the book seems to be absolute; since there is no mention of Dr. Allen's name in his edition, except that in the last paragraph of the preface, he remarks, incidentally, that the work has been prepared 'with the assistance (au der Hand) of a work by Professor J. Adams Allen, of Chicago.' In short, just as Paris bore a stronger testimony than words could express to the beauty of Menelaus's wife, when he carried Helen off—just as Jack Sheppard, by pocketing Lady Barker's diamonds and disregarding her silver plate, paid the highest compliment in his power to the rare stones—so Dr. Elsner's practical eulogy of Dr. Allen's book, leaves nothing to be added in the way of a tribute to its merits."

---

**_Caution to Intending Subscribers to Ziemssen's Cyclopædia of the Practice of Medicine._**

As this great work progresses, it is possible—from some subscribers breaking up their sets, or from other causes—that occasional odd volumes may be offered for sale. Those who desire the complete work are warned against purchasing these, as the Publishers do not engage to supply parts of sets.

Every subscription must be for the entire work. No volumes will be sold separately. WM. WOOD & Co., Publishers,
27 Great Jones Street, New York.

